# A Pathogenesis-Related Protein-Like Gene Is Involved in the *Panax notoginseng* Defense Response to the Root Rot Pathogen

**DOI:** 10.3389/fpls.2020.610176

**Published:** 2021-01-13

**Authors:** Shan Li, Zie Wang, Bifeng Tang, Lilei Zheng, Hongjun Chen, Xiuming Cui, Feng Ge, Diqiu Liu

**Affiliations:** ^1^Faculty of Life Science and Technology, Kunming University of Science and Technology, Kunming, China; ^2^Yunnan Provincial Key Laboratory of Panax notoginseng, Kunming, China

**Keywords:** *Fusarium solani*, *Panax notoginseng*, RNase activity, overexpression, RNAi, defense response

## Abstract

Pathogenesis-related proteins (PRs) are a class of proteins that accumulate in response to biotic and abiotic stresses to protect plants from damage. In this study, a gene encoding a PR-like protein (PnPR-like) was isolated from *Panax notoginseng*, which is used in traditional Chinese herbal medicines. An analysis of gene expression in *P. notoginseng* indicated that *PnPR-like* was responsive to an infection by the root rot pathogen *Fusarium solani*. The expression of this gene was induced by several signaling molecules, including methyl jasmonate, ethephon, hydrogen peroxide, and salicylic acid. The *PnPR-like-GFP* fusion gene was transiently expressed in onion (*Allium cepa*) epidermal cells, which revealed that PnPR-like is a cytoplasmic protein. The purified recombinant *PnPR-like* protein expressed in *Escherichia coli* had antifungal effects on *F. solani* and *Colletotrichum gloeosporioides* as well as inhibited the spore germination of *F. solani*. Additionally, the *in vitro* ribonuclease (RNase) activity of the recombinant PnPR-like protein was revealed. The *PnPR-like* gene was inserted into tobacco (*Nicotiana tabacum*) to verify its function. The gene was stably expressed in T_2_ transgenic tobacco plants, which exhibited more RNase activity and greater disease resistance than the wild-type tobacco. Moreover, the transient expression of hairpin RNA targeting *PnPR-like* in *P. notoginseng* leaves increased the susceptibility to *F. solani* and decreased the *PnPR-like* expression level. In conclusion, the cytoplasmic protein PnPR-like, which has RNase activity, is involved in the *P. notoginsen*g defense response *to F. solani*.

## Introduction

Plants are sessile organisms that are usually subjected to a variety of biotic and abiotic stresses during growth. Specifically, fungal diseases account for 70–80% of all plant diseases. Effector-triggered immunity (ETI) is an important part of plant innate immunity that protects against pathogen infections (Zhang J. et al., 2018). In plants, ETI is established via the recognition of virulence effectors by the corresponding receptor resistance (R) proteins in a specific gene-to-gene manner (Zhang W. et al., 2018). During a pathogen attack, the ETI induction mechanism in plants is activated, leading to the accumulation of pathogenesis-related proteins (PRs) (Mazumder et al., 2013). The accumulated PRs help plants prevent reinfections, resulting in the development of systemic acquired resistance (Zhang et al., 2013).

On the basis of the similarity of their amino acid sequences, structures, serological relationships, and biological activities, the PRs have been grouped into 17 families (Kaur et al., 2017). These PRs have diverse functions, contributing to cell wall rigidification, signal transduction, and antimicrobial activities, and they are mainly expressed in plants as chitinases, glucanases, and thaumatin-like proteins (Farrakh et al., 2018). Many PRs are distributed in plant cell gaps and vacuoles, and their distribution is related to their isoelectric point and the exposure to stress (Jo et al., 2020). The PRs are usually small (10–40 kDa) and mostly acidic. Therefore, they are able to accumulate in intracellular and intercellular spaces.

In plant cells, specific signaling pathways stimulate the defense system, making plant signaling molecules, including some hormones, crucial for the regulation of defense gene expression. Specially, some PR genes are considered as the signature genes of salicylic acid (SA) and jasmonic acid (JA) pathways in model and many crop plants, in which the biotrophic pathogen activates the SA pathway, whereas the necrotrophic pathogen stimulates the JA pathway (Ali et al., 2017). Both abscisic acid and JA up-regulate the expression of ginseng (*Panax ginseng* C.A. Mey) *PR10-1* and *PR10-2* genes to varying degrees (Lee et al., 2012). The moss (*Physcomitrella patens*) *PpPR-10* genes are highly expressed in response to SA (Castro et al., 2016). The application of exogenous methyl jasmonate (MeJA), SA, and ethylene can increase the *NtPR1a* expression level in tobacco (*Nicotiana tabacum*) (Liu Y. et al., 2019). The *ScPR10* transcription level in sugarcane (*Saccharum* spp. hybrid) is reportedly continuously up-regulated in the first 12 h after a MeJA treatment, and *ScPR10* expression is highly induced by SA and MeJA (Peng et al., 2017). In Chilean strawberry (*Fragaria chiloensis*), the relative expression levels of β-1,3-glucanase genes (*FcBG2-1*, *FcBG2-2*, and *FcBG2-3*) and chitinase genes (*FcCHI2-2* and *FcCHI3-1*) are up-regulated by MeJA (Macedo et al., 2017).

Many PRs have antifungal activities. The broad-spectrum antifungal activities of purified maize (*Zea mays*) PR10 can disrupt the conidial germination and hyphal growth of various species, including *Botrytis cinerea*, *Sclerotinia sclerotiorum*, *Fusarium oxysporum*, *Verticillium dahliae*, and *Alternaria solani* (Zandvakili et al., 2017). The *in vitro* antimicrobial activities of most PRs are due to their hydrolytic effects on cell walls, contact toxicity and perhaps their involvement in defense signaling. Moreover, some PRs may function as RNase, suggesting they may degrade fungal RNA during infections (Chen et al., 2013). For example, the recombinant LrPR4 protein expressed in *Escherichia coli* shows RNase activity toward hydrolyzing RNA from *Lycoris radiata* bulbs and has antifungal activity against rice (*Oryza sativa*) blast *Magnaporthe grisea* (Li et al., 2009). The *in vitro* antifungal activity of wheatwin1, a wheat (*Triticum aestivum*) PR4, is due to its RNase ability (Caporale et al., 2004; Bertini et al., 2009). The RNase activity of jelly fig (*Ficus awkeotsang* Makino) PR-4s is correlated to its inhibitory effect on fungal growth (Lu et al., 2012). An earlier investigation confirmed the *in vitro* RNase activity of soybean (*Glycine max*) GmPRP, which is a PR that can inhibit *Phytophthora sojae* mycelial growth (Xu et al., 2014). Another study proved that GbPR10-1 from sea island cotton (*Gossypium barbadense*) adversely affects *V. dahliae* mycelial growth and shows *in vitro* RNase activity (Chen et al., 2013).

*Panax notoginseng* (Burk) F.H. Chen is a perennial herb in the family Araliaceae. Dried *P. notoginseng* roots are important medicinal materials for producing drugs useful for treating cardiovascular diseases. As a component of traditional Chinese herbal medicines, *P. notoginseng* is widely cultivated in Yunnan Province in China. However, the humid environments in which it is grown are suitable for pests and diseases, especially root rot, which is problematic for the sustainable commercial production of *P. notoginseng*. Root rot, which substantially affects the quality and yield of *P. notoginseng* medicinal materials, is mainly caused by the pathogenic fungus *F. solani* (Chen et al., 2015). In an earlier study, we proved that treating *P. notoginseng* roots with exogenous MeJA can significantly improve the resistance of plants to *F. solani*, and the expression levels of many *P. notoginseng* PR genes changed in response to an exogenous MeJA treatment and *F. solani* infection through transcriptome sequencing (Liu D. et al., 2019). To clarify the PR functions that protect plants from *F. solani*, a *PR-like* gene was isolated from *P. notoginseng*. The full-length cDNA sequence of this gene was obtained and named *PnPR-like* (GenBank accession number MT515437). Additionally, the *PnPR-like* expression profile as well as the expression of *PnPR-like* in a prokaryotic system and the subcellular localization of the encoded protein were analyzed. To functionally validate *PnPR-like*, it was overexpressed in tobacco and the hairpin RNA targeting *PnPR-like* was transiently expressed in *P. notoginseng* leaves through the RNAi-mediated gene silencing.

## Materials and Methods

### Plant and Fungal Materials

Sterile tobacco seedlings were cultured in a climate-controlled cabinet and used for genetic transformations. Additionally, 1-year-old *P. notoginseng* plants grown in a greenhouse under double-layered shade nets were used for gene cloning and expression pattern analyses. Prior to their use in experiments, *F. solani*, and *Colletotrichum gloeosporioides* mycelia were maintained on potato dextrose agar (PDA) medium at 4°C.

### Chemical Treatments and Fungal Inoculation

The roots of healthy 1-year-old *P. notoginseng* plants were wounded with scissors, after which the root-dipping method was used to treat plants with SA (200 μM), MeJA (100 μM), ethephon (ETH, 1 mM), or H_2_O_2_ (1 mM) for 30 min. The *P. notoginseng* roots treated with sterile water were used as control samples. The roots were collected at 0, 4, 12, 24, 48, and 72 h after the treatments. Regarding the fungal inoculations, the wounded roots of healthy 1-year-old *P. notoginseng* seedlings were treated with a MeJA solution (100 μM; treatment group) or sterile water (control group) for 30 min. After 24 h, the roots were inoculated with a fresh *F. solani* spore suspension (10^6^ spores mL^–1^) for 30 min. For the two *P. notoginseng* groups, the roots were collected at 0, 4, 12, 24, 48, and 72 h post-inoculation. All samples were immediately frozen in liquid nitrogen and stored at −80°C before the subsequent nucleic acid extraction.

### Rapid Amplification of cDNA Ends (RACE) and Bioinformatics Analyses

The *PnPR-like* unigene sequence determined from the *P. notoginseng* transcriptome sequencing data included the 5′ untranslated region (UTR) and the start codon. Therefore, a 3′ RACE was performed to obtain the full-length cDNA sequence. The gene-specific primers used for the 3′ RACE were designed based on the unigene sequence (Supplementary Table 1). The mRNA was isolated from 100 μg *P. notoginseng* total RNA using the NucleoTrap^®^, mRNA Midi Kit (Macherey-Nagel, Germany). The SMART RACE cDNA Amplification Kit (Clontech, United States) was used to synthesize cDNA and complete the RACE-PCR. The PCR product was cloned into the pMD-18T vector (TaKaRa, Japan) and the resulting recombinant plasmid was inserted into competent *E. coli* DH5α cells. The recombinant clones were analyzed by sequencing. The GenBank blastn tools^1^ were used to assemble the unigene and RACE products. The ORFfinder online tool^2^ was used to search for the open reading frame (ORF) in the assembled sequence. Bioinformatics analyses, including the sequence analysis, multiple sequence alignment, and cluster analysis, were completed as described by Taif et al. (2020).

### Quantitative Real-Time PCR

The *PnPR-like* gene expression patterns in *P. notoginseng* roots during an infection by *F. solani* as well as after a treatment with SA, ETH, MeJA, or H_2_O_2_ were analyzed in a quantitative real-time PCR (qRT-PCR) assay. A gene-specific primer pair was designed based on the *PnPR-like* cDNA sequence (Supplementary Table 1). The *P. notoginseng* actin 2 gene (*PnACT2*; GenBank accession number KF815706.2) was used as an internal reference gene. The qRT-PCR was performed using the ABI Prism 7,500 Sequence Detection System (Applied Biosystems, United States). The qRT-PCR conditions in this study were the same as those described by Liu et al. (2012). The *PnPR-like* expression level in each sample was calculated using the 2^–Δ^ ^Δ^ ^*Ct*^ method. The qRT-PCR analysis was completed with three replications.

### Subcellular Localization

The *PnPR-like* ORF was cloned into the pGEM-T Easy vector (Promega, United States) to produce the pGEM-T Easy-*PnPR-like* plasmid, which was digested with *Nde*I and *Bam*HI. The same restriction enzymes were used to digest the binary expression vector pBIN m-gfp5-ER, which includes the GFP (green fluorescent protein) gene. The *PnPR-like* ORF was incorporated into pBIN m-gfp5-ER using T4 DNA ligase (Promega, United States) to generate the pBIN m-gfp5-ER-*PnPR-like* recombinant plasmid, which included the *PnPR-like-GFP* fusion gene. This plasmid was inserted into *Agrobacterium tumefaciens* EHA105 cells using the CaCl_2_ freeze–thaw method. The transient expression of the *PnPR-like-GFP* fusion gene in onion epidermal cells was analyzed as described by Liu et al. (2018).

### Expression and Purification of the Recombinant PnPR-Like Protein in *E. coli*

The *PnPR-like* ORF was removed from the pGEM-T Easy-*PnPR-like* plasmid by restriction enzyme digestion and then subcloned into the pET-32a vector. The resulting pET-32a-*PnPR-like* plasmid was inserted into competent *E. coli* BL21 cells. The transformed *E. coli* cells were cultured in liquid Luria-Bertani (LB) medium supplemented with ampicillin (50 mg L^–1^) for 12 h at 37°C. When the optical density (at 600 nm) of the bacterial culture reached 0.6, isopropyl β-D-1-thiogalactopyranoside (IPTG, 1 mM final concentration) was added, after which the bacterial culture was incubated at 25°C in a shaker to induce *PnPR-like* expression. Aliquots of the bacterial culture were collected at 2, 4, 6, and 8 h after the *E. coli* cells were induced. The bacterial cells were lysed with lysozyme and then centrifuged. The supernatant and the precipitate were collected and analyzed by SDS-PAGE. The polyhistidine-tagged recombinant PnPR-like protein was purified using a Ni-NTA column (Sangon Biotech, China) as described by Wang et al. (2019).

### Analyses of the RNase and Antifungal Activities of the Recombinant PnPR-Like Protein

The recombinant PnPR-like protein concentration was determined with the Bradford Protein Assay Kit (Sangon Biotech). To further evaluate the PnPR-like activity, fresh *F. solani* and *C. gloeosporioides* mycelia were activated on agar-solidified PDA medium. When the diameter of the fungal colonies reached 2 cm, sterile filter paper disks (0.6 mm in diameter) containing 5, 10, and 20 μg recombinant PnPR-like protein were added around the fungal colonies. As controls, sterile filter paper disks moistened with ddH_2_O and buffer were also placed around the fungal colonies. After a 3-day incubation at 28°C, the *in vitro* antifungal effects of the recombinant PnPR-like protein on *F. solani* and *C. gloeosporioides* were observed. Specifically, three replications of the antifungal assay were analyzed with Photoshop 7.0 to calculate the average mycelial growth inhibition areas (mm^2^) for the two pathogenic fungi. To further observe the inhibitory effect of PnPR-like on the growth of *F. solani*, the spore growth inhibition was also tested. The *F.solani* spores were grown in the presence of recombinant PnPR-like proteins (40 μg/ml) using glucose solution as control. Inhibition of spore germination was evaluated after 8 h at 21°C and was examined under an optical microscopy (Nikon, JPN). Images acquired through the digital photo camera were sized and optimized for contrast and brightness using Photoshop 7.0. The experiment was carried out at least three times independently using the same conditions.

RNase activity of the recombinant PnPR-like was performed using 20 μg protein incubated with 5 μg of extracted RNA from *F. solani*. The reaction was incubated at 37°C for 30 min, the proteins were removed by phenol–chloroform (1:1) extraction and the results were observed on 1.4% agarose gel. RNase Inhibitor (40 U; promega, United States) was used as a control. The value of RNase activity of the recombinant PnPR-like protein was measured by spectrophotometric assay. The reaction mixture contained 20 μg of *F.solani* RNA and 1 μg of recombinant protein. After 30 min incubation at 37°C, the residual not degraded RNA was recovered by an equal volume of 4 M LiCl at 4°C for 3 h, whereas the free oligonucleotides remained in the supernatant. The reaction mixture without incubation contained the same content of *F.solani* RNA, recombinant protein, and 4M LiCl was as a control. The absorbance of the supernatant at 260 nm was read in comparison with the absorbance of control RNA treated as above. One unit of RNase activity (U/mg) was defined as one OD_260_ value adding from the digested RNA by 1 mg protein.

### Generation and Screening of Transgenic Tobacco Plants

The *PnPR-like* ORF fragment was obtained by digesting the pGEM-T*-PnPR-like* plasmid with *Bam*HI and *Eco*R I. The *PnPR-like* fragment was then inserted into the pCAMBIA2300s plant expression vector digested with the same two restriction enzymes. Competent *E. coli* DH5α cells were transformed with pCAMBIA2300s-*PnPR-like*. The transformation was confirmed by PCR. The recombinant plasmid was then inserted into *A. tumefaciens* LBA4404 cells using a CaCl_2_ freeze–thaw method, with transformants detected by PCR. The *A. tumefaciens* cells carrying pCAMBIA2300s-*PnPR-like* were used to transform *N. tabacum* L. cv “Xanthi” leaf disks (Horsch et al., 1985). The T_0_ generation transgenic tobacco plants verified by PCR were grown in a greenhouse to produce the T_1_ and T_2_ generations.

### Analysis of Transgene Expression and Evaluation of the Disease Resistance of T_2_ Transgenic Tobacco

The *PnPR-like* transcription level in T_2_ tobacco lines was analyzed by qRT-PCR. The *PnACT2* gene was used as an internal reference control. Additionally, wild-type (WT) tobacco served as a negative control, whereas the transgenic tobacco line with the highest Ct value was used as a positive control (The expression level in this line was set as 1). In addition, the RNase activity of total protein of *PnPR-like* transgenic tobacco lines was tested. The 0.5 g tobacco leaves of transgenic tobacco and WT were quickly ground into powder with liquid nitrogen, and then 500 μL of protein extract was added with sufficient grinding, which was following the incubation at 4°C for overnight. After centrifuging at 4°C and 12,000 *g* for 30 min, the supernatant was obtained to test the RNase activity by spectrophotometric assay as previously described.

To further functionally characterize *PnPR-like*, four transgenic tobacco lines in which *PnPR-like* was expressed at relatively high levels were examined regarding their resistance to fungal infections. The WT plants were used as controls. Fully expanded tobacco leaves of a uniform size were wounded and then inoculated with 100 μL *F. solani* spore suspension (10^6^ spores mL^–1^). The inoculated leaves were placed on sterile water-soaked filter paper in a foam box, which was then sealed with plastic wrap and maintained under light in an incubator set at 25°C for 1 week. The leaves were collected and the lesions due to the *F. solani* infection were examined. Additionally, the roots of sterile WT and transgenic tobacco plants were wounded and then immersed in the *F. solani* spore suspension (10^6^ spores mL^–1^) for 30 min. The inoculated tobacco plants were grown in Hoagland nutrient solution under light in an incubator set at 25°C for 1 week. The disease symptoms induced by the *F. solani* infection were analyzed.

### Transient Expression of Hairpin RNA Targeting *PnPR-Like* in *P. notoginseng*

Primers specific for *PnPR-like* were designed and then modified at the 5′ end by the addition of the attB adapter sequence (Supplementary Table 1). The PCR product with the attB adapter sequence as well as the RNAi vector pHellsgate 2 were subjected to a BP recombination reaction using the BP Clonase^TM^ II enzyme mix (Invitrogen, United States). The reaction product was inserted into competent *E. coli* DH10B cells, with the transformants selected on agar-solidified LB medium containing spectinomycin (90 mg L^–1^). The recombinant plasmid was subsequently digested with *Xba*I and *Xho*I to confirm the exogenous gene fragments were correctly recombined.

The pHellsgate 2-*PnPR-like* recombinant plasmid and an empty pHellsgate two vector were inserted into separate *A. tumefaciens* EHA105 cells. Positive clones were selected on agar-solidified LB medium supplemented with kanamycin (90 mg L^–1^) and rifampicin (20 mg L^–1^). The cells carrying the empty pHellsgate two vector were used as controls. The *A. tumefaciens* EHA105 clones were added to MGL medium and incubated for 5 h at 28°C in a constant temperature shaker (150 rpm). Young *P. notoginseng* leaves were wounded and placed on filter paper moistened with water. The *A. tumefaciens* cells containing the RNAi vector were added to the *P. notoginseng* leaves at the wound site. The hairpin RNA from pHellsgate 2-*PnPR-like* was transiently expressed in *P. notoginseng* leaves for approximately 24 h in a climate-controlled cabinet set at 25°C, after which a fresh *F. solani* spore suspension (10^6^ spores mL^–1^) was used to inoculate the leaves at the wound site. The infected leaves were collected at 72 h after the inoculation for an examination of the lesions caused by *F. solani*. The samples were frozen in liquid nitrogen and stored at −80°C. Finally, the *PnPR-like* expression level after the transient expression of the hairpin RNA used for RNAi in *P. notoginseng* leaves was analyzed by qRT-PCR.

### Statistical Analyses

Data regarding the relative *PnPR-like* expression levels, the inhibition of fungal growth by the recombinant PnPR-like protein, RNase activity, and the lesion areas are presented herein as the mean ± standard deviation. The data were analyzed with the SPSS software (version 17.0), using Student’s *t*-test to determine the significance of the differences between the treated or inoculated and control samples as well as between the WT and T_2_ transgenic lines.

## Results

### Isolation and Analysis of a *PnPR-Like* Gene From *P. notoginseng*

**FIGURE 1:**
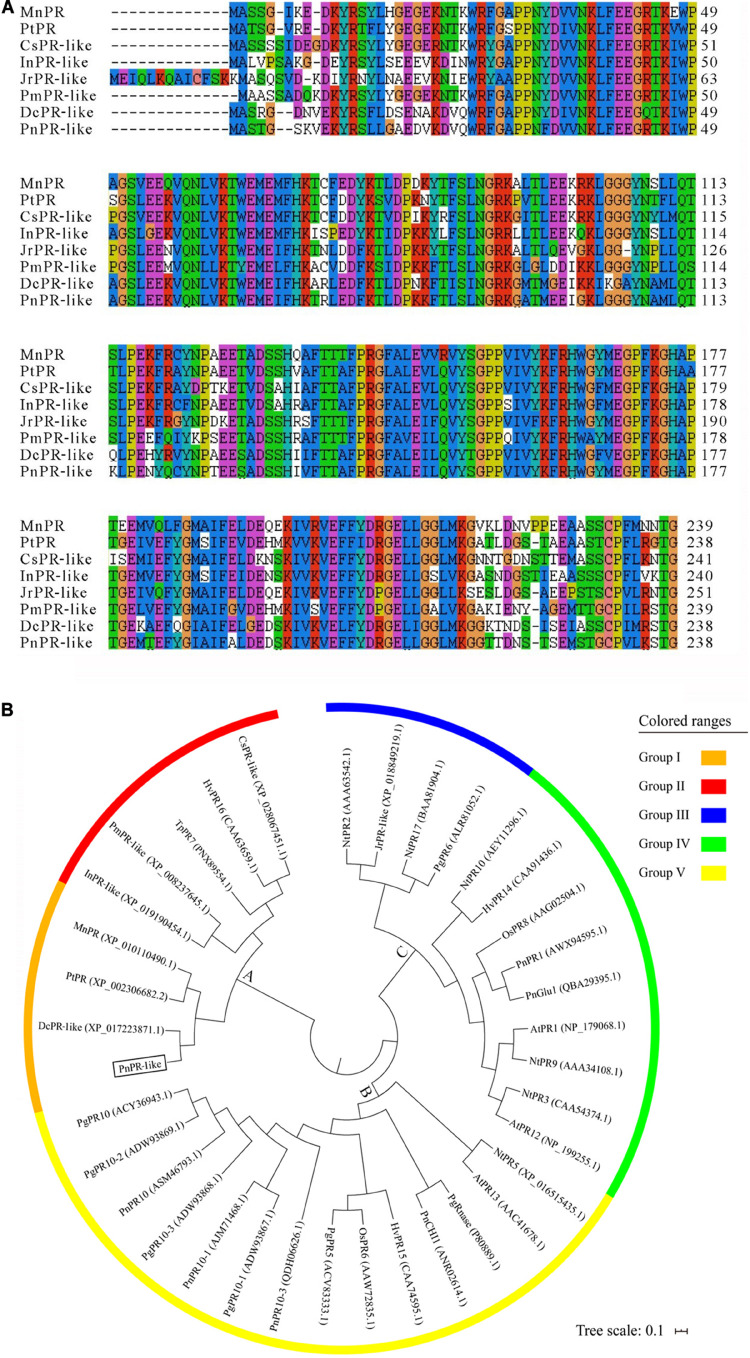
Multiple sequence alignment and phylogenetic tree of PnPR-like and some known PRs (like). **(A)** The multiple alignment of the deduced amino acid sequences of *PnPR-like* and seven homologous sequences were performed with the ClustalW. **(B)** The phylogenetic tree of PnPR-like and 35 known PRs was constructed with Mega seven. The scale bar equals a distance of 10 changes per 100 amino acid positions. The protein sequences are from *M. notabilis* (MnPR, XP_010110490.1), *P. trichocarpa* (PtPR, XP_002306682.2), *C. sinensis* (CsPR-like, XP_028067451.1), *I. nil* (InPR-like, XP_019190454.1), *J. regia* (JrPR-like, XP_018849219.1), *P. mume* (PmPR-like, XP_008237645.1), carrot (DcPR-like, XP_017223871.1), *N. tabacum* (AtPR1, NP_179068.1), *N. tabacum* (NtPR2, AAA63542.1), *N. tabacum* (NtPR3, CAA54374.1), *N. tabacum* (NtPR5, XP_016515435.1), rice (OsPR6, AAW72835.1), *T. pretense* (TpPR7, PNX89554.1), rice (OsPR8, AAG02504.1), *N. tabacum* (NtPR9, AAA34108.1), *N. tabacum* (NtPR10, AEY11296.1), *A. thaliana* (AtPR12, NP_199255.1), *A. thaliana* (AtPR13, AAC41678.1), wheat (HvPR14, CAA91436.1), wheat (HvPR15, CAA74595.1), wheat (HvPR16, CAA63659.1), *N. tabacum* (NtPR17, BAA81904.1), *P. ginseng* (PgPR5, ACV83333.1), *P. ginseng* (PgPR6, ALR81052.1), *P. ginseng* (PgPR10-1, ADW93867.1), *P. ginseng* (PgPR10-2, ADW93869.1), *P. ginseng* (PgPR10-3, ADW93868.1), *P. ginseng* (PgRnase, P80889.1), *P. notoginseng* (PnPR1, AWX94595.1), *P. notoginseng* (PnPR10, ASM46793.1), *P. notoginseng* (PnPR10-1, AJM71468.1), *P. notoginseng* (PnCHI1, ANR02614.1), *P. notoginseng* (PnGlu1, QBA29395.1), *P. notoginseng* (PnPR10, ASM46793.1), and *P. notoginseng* (PnPR10-3, MK238491), respectively.

In this study, a gene encoding a PR-like protein was isolated from *P. notoginseng*. The full-length *PnPR-like* cDNA is 976 bp in length, with a 717-bp ORF, a 96-bp 5′ UTR, and a 163-bp 3′ UTR. The *PnPR-like* ORF encodes a protein with 238 amino acid residues, with a molecular mass of approximately 26.64 kDa and an isoelectric point of about 5.69. A BLASTp analysis indicated that the protein sequence encoded by *PnPR-like* is very similar to the sequences of many PR-like proteins and PRs, including DcPR-like (XP_017223871.1) in carrot (*Daucus carota*), CsPR-like (XP_028067451.1) in *Camellia sinensis*, InPR-like (XP_019190454.1) in *Ipomoea nil*, PmPR-like (XP_008237645.1) in *Prunus mume*, JrPR-like (XP_018849219.1) in *Juglans regia*, MnPR (XP_010110490.1) in *Morus notabilis*, and PtPR (XP_002306682.2) in *Populus trichocarpa*. A multiple sequence alignment of PnPR-like and the homologous sequences confirmed the high homology among these protein sequences (Figure 1A).

In addition to PnPR-like and the above-mentioned 7 homologous proteins, 13 PRs from *Panax* species and 15 other PR family proteins were used to construct a phylogenetic tree. The PRs and PR-like proteins were divided into three clusters (A–C) and further subdivided into five groups. Cluster A included PnPR-like, the two homologous PRs and four PR-like proteins, TpPR7, and HvPR16 (Figure 1B), implying that PnPR-like is highly homologous to PRs. Additionally, PnPR-like, MnPR, PtPR, and DcPR-like formed Group I. The 13 PRs from *Panax* species were unequally distributed among the three clusters. Specifically, the PR10s, including PnPR10, PgPR10, PnPR10-1, PnPR10-3, PgPR10-1, PgPR10-2, and PgPR10-3, as well as PgPR5, PgRnase, and PnCHI1 belonged to Cluster B, whereas PgPR6, PnPR1, and PnGlu1 were included in Cluster C. These phylogenetic relationships suggest that *PnPR-like* encodes a *P. notoginseng* PR.

### *PnPR-Like* Expression Is Responsive to Several Signaling Molecules and Is Up-Regulated During the *F. solani* Infection

**FIGURE 2:**
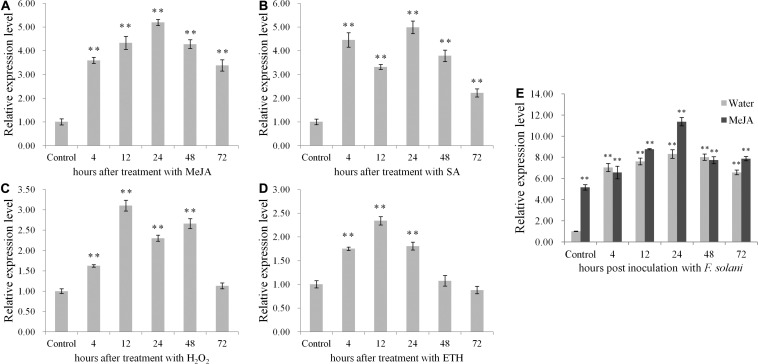
The expression profiles of *PnPR-like* in *P. notoginseng* roots. **(A–E)** The relative expression levels of *PnPR-like* after treatment with four signal molecules including MeJA **(A)**, SA **(B)**, H_2_O_2_ **(C)**, and ETH **(D)** as well as after inoculation with *F. solani* **(E)** were analyzed by qRT-PCR. The roots were collected at 4-, 12-, 24-, 48-, and 72-h post-inoculation, respectively. The *P. notoginseng* roots respectively pretreated with MeJA and sterile water were inoculated with *F. solani* spore’s suspension. Results are showed with the average values calculated from three replicates, and the statistical differences between the treatments or inoculation and the control were analyzed by Student’s *t*-test (**p < 0.01).

The qRT-PCR data revealed the up-regulated expression of *PnPR-like* following treatments with four signaling molecules (Figures 2A–D). Relative to the control level, the *PnPR-like* expression level was highest in the roots after the MeJA treatment, followed by the SA, H_2_O_2_, and ETH treatments. The *PnPR-like* gene was most highly expressed (i.e., 5.2-times higher than the control level) at 24 h after the treatments with MeJA. Regarding the SA treatment, the *PnPR-like* expression level was highest at 24 h (i.e., 5-times higher than the control level). The highest *PnPR-like* expression levels in response to the H_2_O_2_ and ETH treatments (i.e., 3.2- and 2.3-times higher than the control level, respectively) were detected at the 12-h time-point.

After the inoculation with *F. solani*, *PnPR-like* expression increased immediately in the *P. notoginseng* roots pre-treated with MeJA, with an expression level about 5.1-times higher than that in the plants pre-treated with water (Figure 2E). Moreover, the peak *PnPR-like* expression levels in the two groups of *P. notoginseng* roots were detected at 24 h; the expression level in the roots pre-treated with MeJA was about 1.4-times higher than that in the roots pre-treated with water and 11.2-times higher than that in the control roots (uninoculated roots pre-treated with sterile water).

### The PnPR-Like Protein Is Localized in the Cytoplasm

**FIGURE 3:**
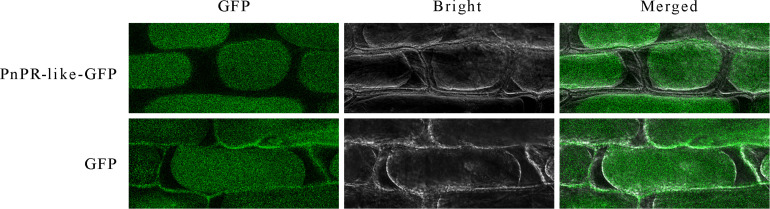
Subcellular analysis revealed the location of *PnPR-like* in the cytoplasm through the transient expression of the *PnPR-like*-GFP fusion gene in the onion epidermal cells. The *PnPR-like*-GFP fusion gene and GFP (control) were transiently expressed in onion epidermal cells mediated by *A. tumefaciens*. The onion epidermal tissues were treated with 20% sucrose solution to induce plasmolysis, and then a confocal laser microscopy was used to display GFP (green fluorescence), bright field (bright), and combination (merged) views, respectively.

The deduced PnPR-like protein lacks a predicted signal peptide and may be an endocrine protein. To investigate the subcellular localization of PnPR-like, a *PnPR-like-GFP* expression cassette was constructed and transiently expressed in onion epidermal cells following *A. tumefaciens*-mediated transformation. An examination with a confocal laser scanning microscope indicated that the GFP signal was specifically distributed in the cytoplasm of onion epidermal cells in which the *PnPR-like-GFP* fusion gene was expressed, whereas it was detected throughout the cells transformed with the empty GFP vector (Figure 3). Thus, PnPR-like appears to be a cytoplasmic protein.

### The PnPR-Like Recombinant Protein Expressed in *E. coli* Has RNase and Antifungal Activities

**FIGURE 4:**
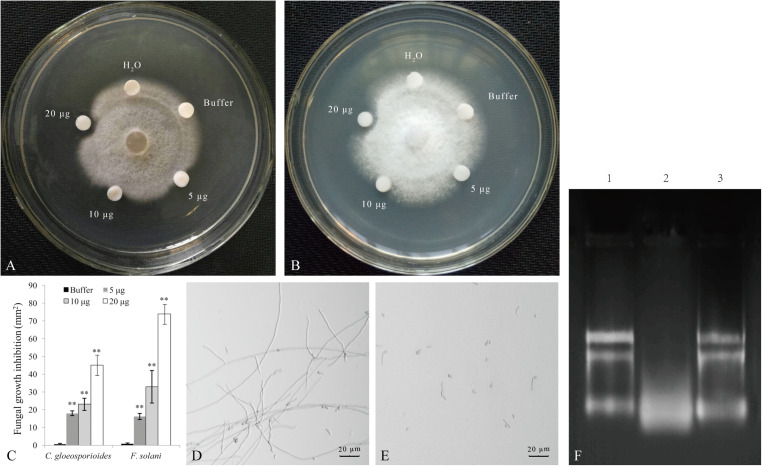
The antifungal assay of the recombinant PnPR-like protein expressed in *E. coli*. **(A,B)** The recombinant PnPR-like protein has evident antifungal activity to *C. gloeosporioides* **(A)** and *F. solani* **(B)**. **(C)** The fungal growth inhibition analysis shows the antifungal activities are increased with the increase of recombinant PnPR-like protein. **(D,E)** Antifungal effect of PnPR-like on *F. solani* spore germination revealed by optical microscopy. *F. solani* spores germinated in the presence of glucose solution **(D)** and PnPR-like **(E)**, respectively. **(F)** RNase activity of the recombinant PnPR-like protein tested against *F. solani* total RNA. The *F. solani* total RNA (lane 1) was incubated with PnPR-like for 30 min (lane 2); The *F. solani* total RNA was incubated with PnPR-like and RNase inhibitor for 30 min (lane 3). Results are showed with the average values calculated from three replicates, and the statistical differences are analyzed by Student’s *t*-test (**p < 0.01).

The SDS-PAGE analysis revealed that the recombinant PnPR-like protein expressed in *E. coli* is about 43 kDa, which is consistent with the expected size. The recombinant protein was purified with a Ni-NTA column and then concentrated with a Millipore ultrafiltration tube. In the subsequent antifungal assay, the recombinant PnPR-like protein significantly inhibited *C. gloeosporioides* and *F. solani* mycelial growth (Figures 4A,B). For both fungi, the mycelial growth inhibition increased as the amount of recombinant PnPR-like protein increased. Moreover, the inhibitory effect of the recombinant protein was greater for *F. solani* than for *C. gloeosporioides* (Figure 4C). To further observe the inhibitory effect of PnPR-like on the growth of *F. solani*, spore growth inhibition was also tested. The Figure 4D showed the growth of *F. solani* in the absence of PnPR-like protein, whereas the effect of PnPR-like is reported in Figure 4E. It is clear that the PnPR-like protein obviously inhibited the germination of *F. solani* spores and the mycelium growth. Moreover, the recombinant PnPR-like protein exhibited *in vitro* RNase activity (Figure 4F), and the tested enzymatic activity was 325 U mg^–1^. RNA degradation caused by 20 μg of PnPR-like protein was clearly visible (lane two, Figure 4F). Meanwhile, its RNase activity was proved to be sensitive to the RNase inhibitor treatment, and no degradation was observed in presence of 20 μg of PnPR-like plus RNase inhibitor (lane three, Figure 4F).

### The Overexpression of *PnPR-Like* in Tobacco Confers a High Level of Resistance to *F. solani*

**FIGURE 5:**
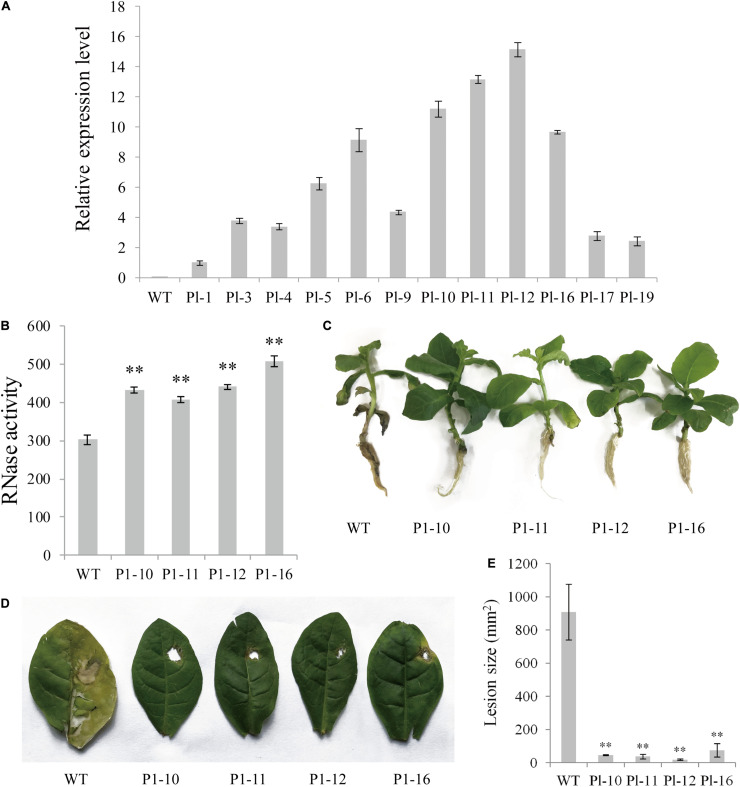
The expression levels, RNase activities, and resistance analyses of T2 generation *PnPR-like* transgenic tobacco. **(A)** The expression levels of *PnPR-like* in the T2 generation of transgenic tobacco were by detected through qRT-PCR, and *PnPR-like* was expressed in all transgenic tobacco plants. The Pl-1, Pl-3, Pl-4, Pl-5, Pl-6, Pl-9, Pl-10, Pl-11, Pl-12, Pl-16, Pl-17, and Pl-19 are the T2 generation transgenic tobacco lines, and WT is the wild-type tobacco. **(B)** The RNase activity of total protein of *PnPR-like* transgenic tobacco and WT tested against *F. solani* total RNA. **(C)** The roots inoculation assay reveals the enhanced resistance of *PnPR-like* transgenic tobacco lines against *F. solani*. **(D)** The leave inoculation assay shows the enhanced resistance of *PnPR-like* transgenic tobacco lines against *F. solani*. **(E)** The lesion sizes in leaves of *PnPR-like* transgenic tobacco lines and WT during *F. solani* infection. The results show the mean of three replicate calculations and statistical differences were analyzed by Student’s *t*-test (**p < 0.01).

The *PnPR-like* gene was inserted into tobacco plants to investigate its biological function related to defense responses. The T_0_ generation transgenic tobacco plants were obtained via a leaf disk transformation method, with 32 positive *PnPR-like* transgenic tobacco plants identified by a PCR analysis. Twelve transgenic tobacco plants were randomly selected to develop T_2_ lines by self-crossing for two generations. There were no visible phenotypic differences between the T_2_ tobacco lines and the WT control. Additionally, qRT-PCR data indicated that *PnPR-like* was stably expressed in 12 analyzed T_2_ transgenic tobacco lines (Figure 5A). More specifically, the *PnPR-like* expression levels were highest in transgenic lines Pl-10, Pl-11, Pl-12, and Pl-16 (Figure 5A). Accordingly, these four lines were used for testing the RNase activity and evaluating the resistance to fungal infections. The total protein of WT and *PnPR-like* transgenic tobacco showed the RNase activity, but the RNase activity of the *PnPR-like* transgenic lines was significantly higher than the WT (Figure 5B).

At 7 days after the inoculation with *F. solani*, the roots of WT tobacco plants were blackened and the leaf chlorosis was severe. In contrast, the roots of the four *PnPR-like* transgenic lines were growing normally, with no blackening or rotting (Figure 5C). Furthermore, the *F. solani* infection resulted in mild chlorosis around the inoculation site of the transgenic tobacco leaves, whereas the WT tobacco leaves had severe chlorotic lesions and were rotting (Figure 5D). The lesions were almost 12-times larger on the WT leaves than on the transgenic tobacco leaves (Figure 5E) at 7 days after the inoculation with *F. solani*. The inoculation assay results suggest that the transgenic tobacco plants were more resistant to *F. solani* than the WT plants. Thus, the overexpression of *PnPR-like* in tobacco confers strong resistance to *F. solani*.

### The Transient Expression of the Hairpin RNA Targeting *PnPR-Like* in *P. notoginseng* Leaves Increases the Susceptibility to *F. solani*

**FIGURE 6:**
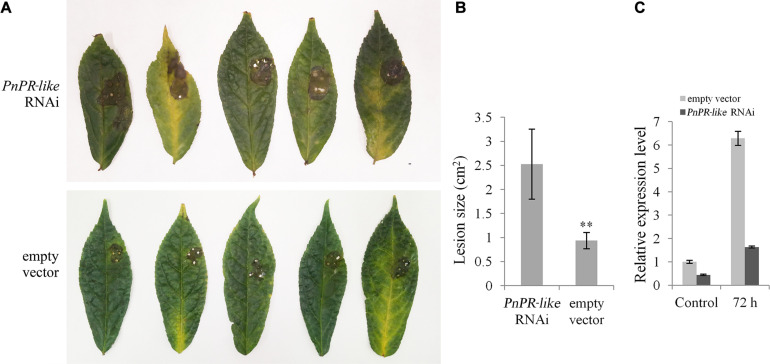
Analysis of the hairpin RNA targeting *PnPR-like* transiently expressed in the *P. notoginseng* leaves. **(A)** The symptoms of *P. notoginseng* leaves after inoculation with *F. solani*, in which the *PnPR-like* RNAi vector and the empty vector was expressed, respectively. **(B)** The lesion size in *P. notoginseng* leaves caused by *F. solani* infection using Student’s *t*-test (**p < 0.01). The transient expression of the hairpin RNA targeting *PnPR-like* increased the susceptibility of *P. notoginseng* leaves to *F. solani*. **(C)** The relative expression levels of *PnPR-like* in the two group of *P. notoginseng* leaves evaluated by qRT-PCR. After inoculation with *F. solani* for 72 h, the *PnPR-like* expression level in the control leaves was about 5-times higher than that in the leaves transformed with pHellsgate 2-*PnPR-like*, which indicates that the transient expression of RNAi construct decreased the expression level of *PnPR-like* in *P. notoginseng* leaves. Results are showed with the average values calculated from three replicates, and the Student’s *t*-test was used to analyze the statistical differences (**p < 0.01).

The pHellsgate 2-*PnPR-like* and the pHellsgate two empty vector were inserted into separate *P. notoginseng* leaves, which were then inoculated with *F. solani*. At 3 days post-inoculation, the infected leaves were withered, yellow, and decayed around the inoculation site (Figure 6A). However, the lesions were significantly larger on the leaves transformed with pHellsgate 2-*PnPR-like* than on the leaves transformed with the empty vector. The average leaf lesion area was 2.5 cm^2^ for the *PnPR-like* RNAi samples, whereas it was less than 1 cm^2^ for the empty vector control samples (Figure 6B). Additionally, the *PnPR-like* expression level in the control leaves was about five-times higher than that in the leaves transformed with pHellsgate 2-*PnPR-like* (Figure 6C). Thus, the transient expression of the hairpin RNA targeting *PnPR-like* increased the susceptibility of *P. notoginseng* leaves to *F. solani*.

## Discussion

Pathogenesis-related proteins are widely distributed in higher plants, wherein they are important for defense responses that protect plant cells from diverse stresses. In this study, a new PR gene, *PnPR-like*, was isolated from *P. notoginseng.* The deduced PnPR-like amino acid sequence is highly similar to the sequences of some known plant PR-like proteins. The PRs are mainly distributed in the intercellular space. However, the deduced PnPR-like protein appears to lack a signal peptide, and its subcellular localization suggests PnPR-like may be an intracellular protein. In the current study, the PnPR-like-GFP fusion protein was detected exclusively in the cytoplasm of onion epidermal cells, implying *PnPR-like* encodes a cytoplasmic protein. In the PR family, PR10 is a class of typical cytoplasmic proteins with no signal peptide (Han et al., 2017). Previous studies proved that GmPR10 (Xu et al., 2014), ZmPR10.1 (Xie et al., 2010), JcPR10a (Agarwal et al., 2012), and *P. notoginseng* PR10-3 (Tang et al., 2019) are localized in the cytoplasm. These intracellular proteins are synthesized by free ribosomes and help mediate cytosolic reactions associated with mitochondria, nuclei, and peroxidases. Some intracellular PRs can bind to ligands, including hormones, biological macromolecules, and active substances with important biological functions, to regulate growth and development as well as defense responses (Mogensen et al., 2002).

The expression of many PR genes is regulated by specific hormone signaling pathways. In *Arabidopsis thaliana*, SA treatments significantly up-regulate *PR1*, *PR2*, and *PR5* expression, but have the opposite effect on the expression of *PR3*, *PR12*, and *PR13* (Seo et al., 2008). The expression of *Brassica juncea* PR genes is responsive to JA. The *BjPR3*, *BjPR4*, and *BjPR12* expression levels reportedly increase following a JA treatment, whereas an abscisic acid treatment down-regulates the transcription of *BjPR1*, *BjPR2*, and *BjPR5* (Ali et al., 2017). An earlier investigation of *Lilium regale* indicated that the stress-related signaling molecules JA, SA, ETH, and H_2_O_2_ up-regulate *PR10-1*/*-2*/*-5*/*-6*/-*7* expression, but down-regulate the expression of *PR10-3/-4*/*-8*/*-9* (He et al., 2014). In the current study, *PnPR-like* expression was up-regulated by MeJA, ETH, H_2_O_2_, and SA to varying degrees. Moreover, *PnPR-like* expression was also induced by an infection of *F. solani*. To effectively defend against pathogens, plants have evolved sophisticated defense mechanisms that “sense” pathogen attacks and activate appropriate defense responses (Ji et al., 2014; Gupta et al., 2015). Additionally, cross-communicating signal transduction networks are formed in plants to regulate the induced defense responses. Specifically, necrotrophic pathogen *F. solani* mainly stimulates JA pathway, whereas the cross-talk among SA, JA, and ETH signaling pathways helps control the defense response of *P. notoginseng* to *F. solani* (Ali et al., 2017; Liu Y. et al., 2019). Moreover, the MeJA regulates the expression of genes encoding PnPR10-3, osmotin-like protein, and β-1,3-glucanase following an *F. solani* infection (Tang et al., 2017; Taif et al., 2020). The activation of *P. notoginseng* PRs, including PnPR-like, is part of a regulatory network mediated by the cross-talk among hormone signaling pathways during an *F. solani* infection; however, the precise regulatory mechanism will need to be more thoroughly characterized.

The PRs are an important part of plant defense systems. Some PRs are hydrolytic enzymes, such as chitinases (NtPR-Q, VpPR4-1, and EuCHIT2) and β-1,3-glucanases (PnGlu1 and HbPR2), that hydrolyze β-1,3-glucans and chitin, which are cell wall components in most higher fungi (Dai et al., 2016; Sunpapao and Pornsuriya, 2016; Dong et al., 2017; Tang et al., 2017). The antifungal activities of PR5 proteins, including thaumatin-like and osmotin-like proteins, result in permeabilized plasma membranes, weakened cell walls, and plasmolysis (Rather et al., 2015). The Ypr6 (proteinase inhibitor), Ypr12 (plant defensin), Ypr13 (plant thionin), and Ypr14 (lipid transfer protein) antimicrobial peptides are usually cysteine-rich peptides with broad-spectrum antifungal activities (Loon et al., 2006; Nawrot et al., 2013). Many PR10 proteins and PR4 protrins have been shown to have RNase activity (Xu et al., 2010; Wang et al., 2011; Yang et al., 2018). The recombinant *Theobroma cacao* PR-10 protein has antifungal activities against the basidiomycetous pathogen *Moniliophthora perniciosa* and the yeast *Saccharomyces cerevisiae*, while also exhibiting *in vitro* and *in vivo* RNase activities (Pungartnik et al., 2009). Two PR10 proteins from ginseng (PgPR10s) have been characterized as RNases (Lee et al., 2012). A recent study proved that recombinant PnPR10-3 functions as an RNase *in vitro* has clear antifungal effects on *Fusarium* species (*F. oxysporum*, *F. solani*, and *F. verticillioides*) (Tang et al., 2019). The RNase activity in *Capsicum chinense* PR-4 protein plays a protective role by degrading RNA of invasive pathogens (Guevara-Morato et al., 2010). The antifungal activity of *T. cacao* PR-4b is directly dependent of its RNase activity (Menezes et al., 2014). In the current study, the recombinant PnPR-like protein had RNase activity *in vitro* and was able to inhibit *C. gloeosporioides* and *F. solani* mycelial growth, meanwhile, the spore germination of *F. solani* was significantly inhibited by the recombinant PnPR-like protein. Thus, in addition to the PR10 and PR4 proteins, some other PRs have RNase activities related to plant defense responses during pathogen infections.

The application of reverse genetics technology involving gene overexpression and gene silencing (e.g., RNAi) has enabled the rapid functional characterization of PR genes. For example, overexpressing the sugarcane *PR10* gene in tobacco enhances the resistance of transgenic plants to *Pseudomonas solanacearum* and *F. solani* (Peng et al., 2017). Transgenic grape (*Vitis vinifera*) lines overexpressing *VpPR10.1* are more resistant to downy mildew caused by *Plasmopara viticola* than WT plants (Ma et al., 2018). Constitutive overexpression of wheat *PR4a* and *PR4b* in transgenic tobacco plants decreased *Phytophthora nicotianae* susceptibility, and the T4 generation plants were found to be significantly more resistant against *P. nicotianae* (Fiocchetti et al., 2007). Overexpression of *VpPR4-1* increased powdery mildew resistance of grape (Dai et al., 2016). Additionally, transgenic tobacco plants overexpressing the *L. regale PR10-5* gene exhibit increased resistance to *F. oxysporum* (Chen et al., 2017). The RNAi-mediated silencing of *PR5* expression was observed to decrease the resistance of cherry tomato (*Lycopersicum esculentum*) to *Alternaria alternata* (Zhai et al., 2018). In another study, fungal colonization significantly increased in mature *PR10*-RNAi transgenic maize kernels inoculated with *Aspergillus flavus* (Chen et al., 2010). In the present study, the *F. solani* infection rate was significantly lower for *PnPR-like* transgenic tobacco than for the WT plants, reflecting the increase in disease resistance induced by the overexpression of *PnPR-like*. To further elucidate the PnPR-like function, *PnPR-like* expression was suppressed via RNAi, which resulted in *P. notoginseng* leaves that were more susceptible to *F. solani* than the WT leaves *in vitro*. These findings clearly indicate that *PnPR-like* is a defense response gene in *P. notoginseng* that protects plants against *F. solani* infections. *Fusarium* species, including *F. oxysporum* and *F. solani*, cause serious diseases in many crops, ornamental plants, and medicinal plants. Therefore, they are very important agricultural pathogens. The best way to sustainably and effectively control plant diseases involves developing disease-resistant cultivars (Moscou and van Esse, 2017). A deeper understanding of the molecular basis of plant disease resistance may enhance the genetic engineering of crop species to overcome the limitations of traditional breeding strategies. The *P. notoginseng* genes encoding PRs, including PnPR-like, are excellent candidate genes for the genetic engineering of plants with increased disease resistance.

## Conclusion

The *PnPR-like* gene isolated from *P. notoginseng* in this study encodes a cytoplasmic protein. Additionally, *PnPR-like* expression is responsive to *F. solani* infections and is induced by four signaling molecules, including MeJA. The recombinant PnPR-like protein has significant *in vitro* antifungal and RNase activities. Moreover, the overexpression of *PnPR-like* results in transgenic tobacco plants that is highly resistant to *F. solani*. In contrast, the RNAi-mediated down-regulation of *PnPR-like* expression increases the susceptibility of *P. notoginseng* plants to *F. solani*. Therefore, *PnPR-like* plays an important role in the *P. notoginseng* defense response to the root rot pathogen. The regulatory effects of hormone signaling pathways on *PnPR-like* expression and the mechanism underlying the PnPR-like RNase activity in defense responses to root rot will be explored more precisely in future studies.

## Data Availability Statement

The datasets presented in this study can be found in online repositories. The names of the repository/repositories and accession number(s) can be found below: NCBI: accession number MT515437.

## Author Contributions

SL: methodology, data curation, experiment execution, and writing-original draft preparation. ZW: methodology and material collection. BT: investigation and software. LZ: validation. HC: conceptualization. XC: supervision. FG: methodology. DL: methodology, supervision, writing-reviewing, and editing. All authors contributed to the article and approved the submitted version.

## Conflict of Interest

The authors declare that the research was conducted in the absence of any commercial or financial relationships that could be construed as a potential conflict of interest.

## Footnotes

1 http://www.ncbi.nlm.nih.gov/

2 https://www.ncbi.nlm.nih.gov/orffinder/
