# Supplementary material for: A Pathogenesis-Related Protein-Like Gene Is Involved in the Panax notoginseng Defense Response to the Root Rot Pathogen
Source: Front Plant Sci. 2021 Jan 13;11:610176. doi: 10.3389/fpls.2020.610176 (PMC7838351; doi:10.3389/fpls.2020.610176)
Supplement: Supplementary file 1 [file Table_1.DOCX]

Supplementary Material

# Supplementary Tables

Table S1 The gene-specific primers used for PCR

| Target gene | Primer sequences | TM (°C) |
| --- | --- | --- |
| *PnPR like*  (RACE) | OR: 5′TCAGTGCTACAATCCGACCGAGGAATC3′ | 70 |
| *PnPR like* | QF: 5′ATCAGTGCTACAATCCGACCGA 3′ | 62 |
| (qPCR) | QR: 5′TGGCAATCCCATAAAACTCTGTC 3′ | 61 |
| *PnACT2* | QF: 5′ TCCAAGGGTGAATATGATGAATCG 3′ | 63 |
| (qPCR) | QR: 5′ AACCTCTCCAAAGAGAATTTCTGAGT 3′ | 61 |
| *PnPR like* | OF: 5′ GGATTCCCAAATAACAATACTCTCCACAC 3′ | 56 |
| (Transgenic tobacco screening)  *PnPR like*  (RNAi vector primers with attB) | OR: 5′ GAATTCCGTCCCGCTCCAAGATAGTC 3′  OF:5′GGGGACAAGTTTGTACAAAAAAGCAGGCTGC GCTCCATCCTCAGTACCAGCACAAA 3′  OR:5′GGGGACCACTTTGTACAAGAAAGCTGGGTCT  TCTTCAGTAGGCACCACCGTGAAT 3′ | 57  58  59 |
